# Secretory Protein mRNA Finds Another Way Out

**DOI:** 10.1371/journal.pbio.0050330

**Published:** 2007-12-04

**Authors:** Kira E O'Day

As far as molecular celebrity goes, messenger RNAs (mRNAs) no longer command the spotlight, especially compared with the smoking hot gene-regulating microRNAs or the Piwi-interacting RNAs, with their steamy role in spermatogenesis. As the intermediary between the gene blueprint and the protein end product, mRNAs certainly play a role of fundamental importance, but it's sort of old hat. The path from DNA to mRNA to protein has been established for over 40 years now. After being transcribed from DNA, mRNA is processed: its introns are spliced out, a guanosine cap is added to one end, and a poly-adenosine tail is hooked onto the other. Then, provided it's been processed, it is grabbed by a transcription export (TREX) protein complex and shunted through a nuclear pore into the cytoplasm for translation. But now, new evidence suggests that not all mRNAs follow this tired old script.

It turns out that mRNAs that encode many secretory proteins do indeed have additional functions, as Alexander Palazzo, Tom Rapoport, and their colleagues report in a new study. While many proteins are created and destroyed within the same cell, others are secreted. One property that differentiates secretory proteins from cell-bound proteins is their signal sequence, which recruits protein complexes that grab the new protein while it's still being translated and targets it to the endoplasmic reticulum (ER), where it will be processed for eventual secretion.

Although the most important feature of signal sequences is that they contain a stretch of hydrophobic amino acids, there are still a couple of mysteries surrounding them. For example, it's not clear why signal sequences in human secreted proteins tend to be rich in leucine and poor in isoleucine, even though both of these amino acids are similarly hydrophobic. Another oddity is that the nucleotides encoding signal sequences are conserved across species to a greater extent than might be expected, given that the only requirement for the signal sequence is a hydrophobic stretch of amino acids. This conservation suggests that in addition to encoding the signal sequence, the signal sequence coding region (SSCR) portion of an mRNA might have an alternative function.

While determining what—if any—function the mRNA SSCR might have, Palazzo et al. made the surprising discovery of an alternative pathway out of the nucleus that doesn't require introns or a guanosine cap, bypasses the TREX complex, appears to occur in all vertebrates, and is specific to mRNAs that encode secretory proteins. By injecting mRNAs into the nucleus or by synthesizing mRNAs from DNA introduced into cells and following their export from the nucleus, the authors demonstrated that SSCR-dependent export of mRNAs did not require the transcript to have introns or a guanosine cap, properties thought to be necessary for efficient nuclear export.

When the authors injected RNA containing a fragment of a secretory protein with an SSCR but no introns into cell nuclei, they found that the translated polypeptide could be detected in the ER, indicating that the mRNA had been successfully exported from the nucleus and the resulting polypeptide targeted to the ER. To figure out if the mRNA was being exported using the TREX complex, the authors used RNA interference—a technique that abolishes gene expression—to deplete key components of the complex, and they found no difference in nuclear export. However, as in the intron splicing–dependent export pathway, an additional export factor, TAP, is required. Interestingly, the SSCR even enhances the export of intron-containing mRNAs.

To find out what makes the SSCR so special, the authors conducted a large-scale sequence analysis of various genomes of organisms ranging from bacteria to humans. What they found was that the SSCRs in all eukaryotes have far fewer adenine nucleotides when compared with other translated portions of the genome, with long stretches of sequence containing no adenines at all. These adenine-free stretches were longer in vertebrates than in invertebrates. The authors found a few factors that account for adenine deficiency in the SSCR: as a group, hydrophobic amino acids, which are enriched in the signal sequence, have codons that contain few adenines. And it was these adenine-deficient amino acids with similar hydrophobic tendencies that were favored. One example of this tendency was the bias for leucines over isoleucines noted above—fewer leucine-encoding codons contain an adenine than do isoleucine-encoding codons. Lastly, within each hydrophobic amino acid there was a bias toward codons lacking adenine (this last bias was seen only in vertebrate SSCRs). The authors' analysis suggests that over the course of evolution, there was a strong selection against adenines, likely caused by some requirement of the nucleotide sequence rather than of the encoded amino acid sequence. They confirmed this by changing nucleotides in mRNA SSCRs to adenines and found that these mRNAs were exported from the nucleus with considerably less efficiency.

What might be the purpose of having a separate nuclear transport pathway for proteins destined to be secreted from the cell? It could provide a means of regulating the export and thus the translation of the entire class of secretory protein–encoding mRNAs. And how might the SSCRs be recognized and trigger mRNA nuclear export? The authors favor models in which an export-mediating protein might recognize the lack of adenines in the SSCR and bind to it, or the adenine-lacking portion of the SSCR might fold into a conformation that would attract an export factor, such as a TAP-binding protein. The discovery of an SSCR-mediated alternative nuclear export pathway explains the mysterious amino acid bias in signal sequences—not only does the SSCR act at the amino acid level by targeting proteins to the ER, but it also has an additional, earlier role at the nucleotide level. It also suggests a novel link between mRNA nuclear export and ER membrane targeting—placing the journeyman mRNA back into the limelight and suggesting a number of interesting areas for further exploration, such as whether there are RNA elements located within other coding regions with similar double roles.

